# Dural Sinus Arteriovenous Malformation in the Fetus. Case Report and Discussion of the Literature

**DOI:** 10.3390/diagnostics11091651

**Published:** 2021-09-09

**Authors:** Vlasta Fesslova, Anna Maria Colli, Simona Boito, Isabella Fabietti, Fabio Triulzi, Nicola Persico

**Affiliations:** 1 Center of Fetal Cardiology, IRCCS Policlinico San Donato, 20097 Milan, Italy; 2 U.O. Cardiologia, Fondazione Ca’ Granda, Ospedale Maggiore Policlinico, 20121 Milan, Italy; Anna.Colli@policlinico.mi.it; 3 Fetal Medicine and Surgery Service, Department of Clinical Sciences and Community Health, University of Milan, 20121 Milan, Italy; Simona.Boito@policlinico.mi.it (S.B.); Isabella.Fabietti@policlinico.mi.it (I.F.); Nicola.Persico@policlinico.mi.it (N.P.); 4 Department of Radiology, Fondazione Ca’ Granda, Ospedale Maggiore Policlinico, 20121 Milan, Italy; Fabio.Triulzi@policlinico.mi.it

**Keywords:** fetal echocardiography, fetal ultrasound, fetus, arteriovenous malformations, dural sinus malformation

## Abstract

Sonographic findings of cerebral arteriovenous malformations in the fetus are uncommon and usually regard aneurysm of the Galen vein. Outcome of arteriovenous malformations is usually severe. We report a case of a fetus at 21 weeks’ gestation with a rarer arteriovenous malformation, referred to us for echocardiography on account of a suspicious cardiomegaly at obstetrical scan. Upon examination, we found cardiomegaly, together with an associated moderate tricuspid regurgitation, however, there were no clear features of tricuspid dysplasia. Considering an unusually dilated superior vena cava, we found via color Doppler imaging a systodiastolic flow at Color Doppler progressing. Subsequent MRI of the central nervous system determined the localization in the sinus dura mater. Due to an already evident hemodynamic impact, the parents opted for the termination of the pregnancy. Autopsy confirmed a voluminous arteriovenous malformation of the transverse sinus of the dura mater, severe cardiomegaly, mainly of the ventricles, and hypoplasia of the lungs.

## 1. Introduction

Sonographic findings of cerebral arteriovenous malformations in the fetus are uncommon and usually indicate aneurysm of the Galen vein. The outcome of arteriovenous malformations is usually severe, especially when found early in fetal life, and surgical treatment is not always a satisfactory option. Fetal ultrasound can properly assess the global fetal organs situation, and fetal echocardiography can estimate the cardiac anatomy even in complex defects. However, there may be some cases in which the final diagnosis is rather difficult, mainly in the case of unusual anomalies.

## 2. Case Report

A 31-year-old woman, healthy, gravida 3, para 1 (one previous cesarean section for fetal distress in labor), was referred for fetal echocardiography at 21.2 weeks’ gestation (w.g.) for suspicious cardiomegaly at the routine obstetric scan.

First trimester combined screening (maternal age, nuchal translucency, free β-hCG, and PAPP-A) showed a low risk for common trisomies; nuchal translucency was 1.1 mm. Cell-free DNA testing for trisomy 21, 18, and 13 also returned a low-risk result and showed a normal male karyotype; therefore, the patient opted against an invasive diagnostic procedure.

At fetal echocardiography, a male fetus was in breech position, with normal amniotic fluid. The heart was in the left hemithorax, in situs viscerum solitus. The cardiothoracic ratio was increased (0.6).

Abdominal vessels were normal. The venous pulmonary returns were normal. Inferior caval vein was normal, while superior caval vein seemed dilated. There were two atria and two ventricles, with normal atrioventricular and ventriculoarterial connections. The left atrium was of normal size, with two pulmonary veins draining in; foramen ovale was of a normal size, with right-to-left shunt. The right atrium was dilated, as well as the right ventricle (RV), which presented increased parietal thickness. The tricuspid valve was inserted to the crux cordis, not apparently dysplastic, but presenting a moderate-discrete tricuspid regurgitation, holosystolic, with a flow at color Doppler overpassing the middle portion of the atrium. The RV outflow tract and pulmonary artery were of normal size, with a normal anterograde flow and normal continuity to the duct. No reverse flow through the pulmonary valve or in the duct was evident.

Left-sided structures as well as the heart rhythm were normal. No pericardial effusion or ascites were seen.

**Figure 1:**
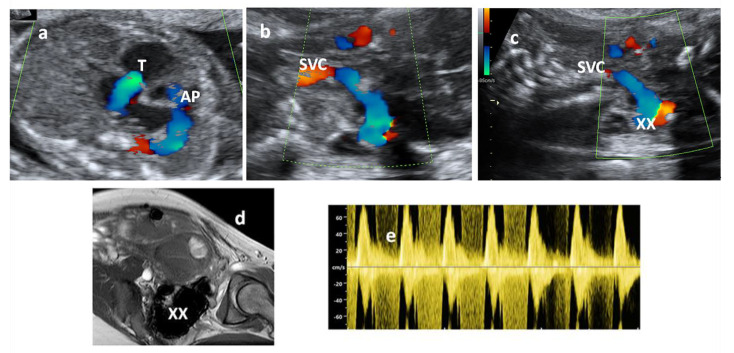
(**a**) Fetal echocardiography—showing tricuspid regurgitation (T) and normal pulmonary artery anterograde flow (AP); (**b**,**c**) Color Doppler flow of the superior caval vein (SVC) proceeding toward the head to an enlarged zone (XX); (**d**) MRI image of the aneurysmatic occipital area (XX); (**e**) Echo Doppler showing systodiastolic flow in this area.

The superior caval vein was more dilated with respect to the inferior caval vein and could be followed up to the cranial portion with a turbulent flow, which reached the occipital portion of the head, with a systodiastolic high velocity flow toward an apparent aneurysmatic enlargement (size circa 30 × 20 mm) (Figure 1a–e and Supplementary Video).

Supplementary Video showing the flow (via color Doppler imaging) passing from superior caval vein (SVC) to the lateral part of the head.

An unusual CNS aneurysmatic formation was suspected, and the woman was sent for a second level obstetrical scan that confirmed the presence of an aneurysmatic area; a CNS MRI examination was therefore performed, reporting a voluminous expansive lesion at the right temporo-parieto-occipital level (sized 30 × 22 mm), with a diagnostic conclusion of a dural sinus malformation (DSM) (Figure 1d).

The couple was informed of a potential negative evolutive impact of the lesion on the course in utero and after birth, considering an early hemodynamic involvement with cardiomegaly, and the couple opted for the termination of the pregnancy.

Postmortem findings confirmed the presence of a voluminous arteriovenous malformation of the transverse sinus of the dura mater, severe cardiomegaly, mainly of the ventricles, and hypoplasia of the lungs. Chorionic villi showed local thrombotic vasculopathy.

## 3. Discussion

Our case presented a rare anomaly—an arteriovenous malformation of the sinus dura mater with an early impact on the heart, with cardiomegaly and tricuspid regurgitation.

The findings at fetal echocardiography were potentially confusing if we had considered only the intracardiac features of cardiomegaly and tricuspid regurgitation. The aspect of tricuspid valve, without a significant dysplasia and with a normal anterograde pulmonary flow, was equivocal. The observation of the dilated superior caval vein pointed toward a different situation: in the differential diagnosis, we had to consider the anomalous pulmonary venous return—excluding it due to evidence of normal drainage of the pulmonary veins into the left atrium, and therefore, the initial suspicion of the operator was an arteriovenous aneurysmatic CNS anomaly, such as, for example, the aneurysm of the Galen vein. However, the localization of the aneurysm was more lateral– and the MRI determined that it was concerning the dura mater.

From the literature, it is known that these anomalies make part of the dural arteriovenous shunts–fistulae and can be separated into the infantile and adult types [1]. They represent less than 2% of miscellaneous vascular malformations and have variable evolution and prognosis [2]. They may have an evolutive character, leading to thrombosis and to a neurological impairment due to abnormal sinus venous drainage [2,3] as well as to death.

A classification system proposed by Borden et al. [2] unifies and organizes spinal and cranial dural arteriovenous fistulous malformations (AVFMs) into three types based upon their anatomical similarities: type I, which drain directly into dural venous sinuses or meningeal veins; type II, which also have retrograde drainage into subarachnoidal veins; and type III malformations, which drain into subarachnoidal veins and do not have dural sinus or meningeal venous drainage. The arterial supply in each of these types is derived from meningeal arteries. A subclassification divides the cases according to the number of fistulas—a single fistula or multiple fistulas. The type 1 malformations are often asymptomatic but can present cranial nerves deficit and radiculopathies. They do not usually present with hemorrhage and have a high rate of spontaneous thrombosis, are considered benign, and require treatment (Transarterial embolizazione is usually required only in the case of cavernous malformations). On the contrary, type 2 and 3 AVFMs usually cause more serious problems, such as bleeding and venous hypertension, and require treatment with thrombosis or ligation of the draining vein, often by a combined surgical–endovascular approach. The dural malformations that present with hemorrhage have a high rate of re-bleeding or a progressive neurological deficit [2].

In adults, the dural arteriovenous malformations may be asymptomatic, and other classifications were proposed based upon the direction of the dural sinus drainage [4] In order to assess the stratification risk for intracranial hemorrhage.

Barbosa et al. [3] published a review of 52 cases of arteriovenous dural shunts in children (Based upon the data from a neurovascular databank between 1985 to 2003) and analyzed the features and outcome of 30 children with dural sinus malformations (DSMs). These cases had a male sex prevalence of 2:1. Around a third of the cases were managed conservatively, without an endovascular treatment, but with a neurological impairment in two thirds of them. Out of the other patients that underwent embolization, a few of them had a relevant neurological outcome. On the whole, in this series, almost half of the cases had an unfavorable neurological outcome, and 11 of the patients died. The authors describe the evolution toward total or partial spontaneous thrombosis before the age of 2 years that compromised normal cerebral venous drainage.

Equally, other reports describe a variable outcome in pediatric cases, with the lesion progressively decreasing in time in some of them, but presenting in others with cardiac failure and the necessity of endovascular embolizations with variable neurological impact [5,6].

In fetuses, knowledge regarding the cause, evolution, and prognosis of DSMs is still limited [7].

Some isolated prenatally diagnosed cases have been reported—found both by ultrasound and by MRI, which are considered to be very useful in terms of the definition and localization of the malformation [8,9,10]. Laurichesse et al. [11] reported on five cases diagnosed by ultrasound and confirmed by MRI, with thrombosis of the dural sinuses that regressed at least partially in three without a hemodynamic impact. Based upon these findings, the authors suggest a noninvasive treatment after birth in similar cases. Equally, a spontaneous prenatal thrombosis was described by other authors [12].

Other prenatal cases had variable outcomes: Komiyama [8] reported on two cases, both presenting with signs of cardiac failure toward the end of pregnancy, needing a cesarean section and a subsequent postnatal endovascular treatment, which was necessary to be repeated, but with both cases having moderate developmental delay, but one with hydrocephalus treated by ventriculo-peritoneal shunt. In some cases, it was reported that the aneurysmatic formation tended to increase, or, vice versa, to produce spontaneous thrombosis and major hemodynamic impact leading at times to death [3,12].

Usually, the prenatal detection of DSM by ultrasound occurs more frequently in the third trimester, and more rarely in the second trimester. Our case was actually one of those found more early, at 21 weeks’ gestation.

Another study presented a report on 13 cases of fetal DSM [13], which were also evaluated by MRI, in which the outcomes were variable—in 10/13 fetuses that continued pregnancy, there was a progressive thrombosis of the mass, which regressed in seven of them, but led to hydrocephalus in one and to atrophy of the frontal lobes in another one. In other cases, postnatal frontal necrosis occurred—as a consequence of the venous imbalance. These findings illustrate a variable and potentially negative outcome of the cases affected. The authors underline the usefulness of MRI, which was also found to be important in our case. Postnatal treatment in these fetal cases included endovascular embolization or derivation in the case of hydrocephalus.

As for the diagnosis, other authors also agree with the usefulness of MRI imaging in utero [14,15]. Mainly, Hart et al. [15] reported a large MERIDIAN study on in utero MRI, considered to be an optimal tool to identify brain anomalies, though without the possibility of predicting developmental outcome.

Treatment by endovascular embolization or surgery is a first line option, but it presents high risk in some cases and is not always resolutive [16,17,18,19].

However, some torcular types of DSM may have a more favorable outcome, both in utero and postnatally [20,21], while DSMs with a giant pouch with a dilation of the transverse and superior sagittal sinus, frequently thrombosed, usually need a surgical excision and present venous hypertension [22].

Our case is particularly interesting due to its early cardiovascular involvement, evident with cardiomegaly and tricuspid regurgitation. Neither the fetal study on DSM nor other authors referred to above describe so specifically an early cardiac impact; however, another study reports on cardiac findings in 11 fetuses with Galen vein and other cerebral arteriovenous malformations, all with tricuspid regurgitation, RV dysfunction, and cardiomegaly, with only five surviving [23].

Effectively, the features of our case were similar to the relevant cardiovascular involvement—high output failure—that occurs in the aneurysm of Galen vein and other cerebral cardiovascular anomalies, with a poor outcome. Jhaveri et al. [24] reported on nine fetuses with similar features of RV dilation and tricuspid regurgitation as in our case, but also with LV dysfunction and with severe brain damage; only four of five infants that underwent postnatal endovascular neurosurgical intervention survived in the long term. A similar negative outcome was reported by another fetal report [25]. Another paper [26] reported on 12 neonates with aneurysm of the Galen vein, all with high output failure, treated by endovascular procedure, with success only in five patients, while the remaining seven patients died of refractory cardiac failure, multiorgan failure, and severe brain damage.

On the other hand, a previous retrospective multicenter study on 49 fetal cases, followed over a 17-year period, stated that 52.9% of the cases that did not undergo termination were alive and free of adverse sequelae; poor outcome was associated with tricuspid regurgitation and a larger volume of the malformation [27]. Apparently, there is still the possibility of a more favorable evolution: in this multicenter series, only 10.2 % of cases showed progression of the lesion.

Our case differs from the reported cases by different localization at MRI, with MRI findings that are easily obtainable [9,15,25], but it seemed to have a similar severe prognosis of evolving heart failure and neurological damage, since it occurred at such an early gestational period.

## Data Availability

The data regarding the study are available in the Fetal data base of the Center of Fetal Cardiology of the IRCCS Policlinico San Donato.

## Supplementary Materials

https://www.mdpi.com/article/10.3390/diagnostics11091651/s1, Supplementary Video.
